# A model to estimate effects of SNPs on host susceptibility and infectivity for an endemic infectious disease

**DOI:** 10.1186/s12711-017-0327-0

**Published:** 2017-06-30

**Authors:** Floor Biemans, Mart C. M. de Jong, Piter Bijma

**Affiliations:** 1 Quantitative Veterinary Epidemiology Group, Wageningen University and Research, Wageningen, The Netherlands; 2 Animal Breeding and Genomics Centre, Wageningen University and Research, Wageningen, The Netherlands

## Abstract

**Background:**

Infectious diseases in farm animals affect animal health, decrease animal welfare and can affect human health. Selection and breeding of host individuals with desirable traits regarding infectious diseases can help to fight disease transmission, which is affected by two types of (genetic) traits: host susceptibility and host infectivity. Quantitative genetic studies on infectious diseases generally connect an individual’s disease status to its own genotype, and therefore capture genetic effects on susceptibility only. However, they usually ignore variation in exposure to infectious herd mates, which may limit the accuracy of estimates of genetic effects on susceptibility. Moreover, genetic effects on infectivity will exist as well. Thus, to design optimal breeding strategies, it is essential that genetic effects on infectivity are quantified. Given the potential importance of genetic effects on infectivity, we set out to develop a model to estimate the effect of single nucleotide polymorphisms (SNPs) on both host susceptibility and host infectivity. To evaluate the quality of the resulting SNP effect estimates, we simulated an endemic disease in 10 groups of 100 individuals, and recorded time-series data on individual disease status. We quantified bias and precision of the estimates for different sizes of SNP effects, and identified the optimum recording interval when the number of records is limited.

**Results:**

We present a generalized linear mixed model to estimate the effect of SNPs on both host susceptibility and host infectivity. SNP effects were on average slightly underestimated, i.e. estimates were conservative. Estimates were less precise for infectivity than for susceptibility. Given our sample size, the power to estimate SNP effects for susceptibility was 100% for differences between genotypes of a factor 1.56 or more, and was higher than 60% for infectivity for differences between genotypes of a factor 4 or more. When disease status was recorded 11 times on each animal, the optimal recording interval was 25 to 50% of the average infectious period.

**Conclusions:**

Our model was able to estimate genetic effects on susceptibility and infectivity. In future genome-wide association studies, it may serve as a starting point to identify genes that affect disease transmission and disease prevalence.

## Background

Infectious diseases in farm animals affect animal health, decrease animal welfare and can affect human health [1]. Infectious diseases also cause economic losses due to disease-related costs, treatment costs, costs for prevention measures, and reduced production [2]. Bacterial infections are often treated with antibiotics, which can lead to antibiotic-resistant bacteria [3]. Viral infections can be prevented with vaccination, which can lead to vaccine escape strains [4, 5]. Thus, it is highly desirable to search for additional ways to fight transmission of infectious diseases. One such approach consists of selecting and breeding host populations for desirable traits regarding infectious diseases [6].

Two main sets of host traits affect transmission of infectious diseases: host susceptibility and host infectivity. Susceptibility is the relative risk of an individual to become infected when exposed to a typical (average) infectious individual or (for infectious diseases transmitted via the environment) the infectious material excreted by a typical infectious individual. Infectivity is the relative propensity of an infected individual to infect a typical (average) susceptible individual.

Studies that investigate host genetic effects related to infectious diseases generally focus on host disease status, and link this to the genotype of the host [7, 8]. By linking own disease status to own genotype, only genetic effects on susceptibility are captured and variation in exposure of susceptible individuals to infectious herd mates is ignored, which may limit the accuracy of estimates of genetic effects on susceptibility. Moreover, there is evidence that genetic variability in infectivity exists as well. Variability in infectivity is found in, for example, super-shedders, i.e. individuals that shed many more infectious units than the average individual in the population [9]. This variability in shedding was found among individuals infected with the “same” pathogen and, thus could be due to host genetic differences.

A host genetic effect on infectivity is an example of an indirect genetic effect (IGE) [10, 11], which is a heritable effect of one individual on the phenotype of another individual [12]. IGE can have profound effects on the rate and direction of evolution by natural selection and on response to selective breeding [10, 12–14]. Thus, genetic effects on infectivity can be used for genetic improvement of populations that suffer from infectious diseases [10, 15] but its use requires different breeding strategies [10, 12, 16]. To design optimal breeding strategies, it is essential to first quantify the genetic effects on infectivity.

Genome-wide association studies (GWAS), in which effects of single nucleotide polymorphisms (SNPs) on a phenotype are estimated, are a common way to quantify genetic effects. To estimate effects of SNPs on susceptibility and infectivity, a generalized linear model with a complementary log–log link function can be used. This model has been applied to data on the final disease status of individuals after an epidemic disease [17, 18], but many diseases are endemic. Furthermore, it is likely that the quality of the estimates improves when data on individual disease status are recorded over multiple (short) time intervals during the infection chain in a population. Each interval can then be seen as an incomplete epidemic, in which only a fraction of the susceptible individuals become infected. For each interval, the infectious individuals to which a susceptible focal individual is exposed are known. Thus, more information on who infected whom and on the rate of infection is available, compared to information on the final disease status only, which is expected to improve the quality of the estimates for host genetic effects on susceptibility and infectivity [19].

Given the potential importance of genetic effects on infectivity, we set out to develop a model to estimate the effects of SNPs on both host susceptibility and host infectivity for an endemic disease. The model accounts for variation among susceptible individuals in exposure to infectious herd mates and for the genotypes of those herd mates. To evaluate the quality of the SNP effects estimated by the model, we simulated an endemic disease and recorded data on individual disease status multiple times during the endemic. We quantified bias and precision of the estimates for different sizes of SNP effects, and identified the optimal recording interval.

## Methods

### Transmission model

Our objective was to develop a model to estimate the effect of single SNPs on disease transmission. Thus, we considered a genetically heterogeneous population of diploid individuals, with one locus for the susceptibility effect, *γ*, and one locus for the infectivity effect $\varphi$. The susceptibility locus had two alleles, allele *G* with value *γ*_*G*_ and allele *g* with value *γ*_*g*_. The infectivity locus also had two alleles, allele *F* with value $\varphi_{F}$ and allele *f* with value $\varphi_{f}$. We assumed additive allele effects on the log-scale, by simulating effects as multiplicative on the original scale such that model terms could be formulated as allele counts within individuals [17]. Thus, susceptibility values were *γ*_*GG*_ = *γ*_*G*_ *γ*_*G*_ = *γ*_*G*_^2^ for genotype *GG, γ*_*Gg*_ = *γ*_*gG*_ = *γ*_*G*_ *γ*_*g*_ for genotype *Gg*/*gG,* and *γ*_*gg*_ = *γ*_*g*_^2^ for genotype *gg*. Likewise, infectivity values were $\varphi_{FF}$ = $\varphi_{F}^2$ for genotype *FF,* $\varphi_{Ff} = \varphi_{fF} = \varphi_{F} \varphi_{f}$ for genotype *Ff*/*fF,* and $\varphi_{ff}$ = $\varphi_{f}^2$ for genotype *ff*. Note that multiplicative allele effects on the original scale introduce dominance on the original scale. Because the value for the heterozygote is lower than the average value of both homozygotes, i.e. *γ*_*Gg*_ < 0.5(*γ*_*GG*_ + *γ*_*gg*_), the dominance is negative (see “Discussion” section).

An endemic disease was modelled with a stochastic compartmental susceptible-infected-susceptible-model (SIS-model). In a SIS-model, two events can occur: infection of a susceptible individual and recovery of an infected individual. Infected individuals were immediately infectious and recovered individuals were immediately susceptible again. Thus, no lasting immunity to disease was assumed. Events (infection and recovery) occurred randomly with a probability per unit of time, depending on model parameters and disease status of individuals in the population.

In a genetically homogeneous population, the expected rate with which susceptible individuals become infected equals $\frac{dS}{dt} = \beta I\frac{S}{N}$, where *I* is the number of infectious individuals, *S* the number of susceptible individuals, and *S* + *I* = *N*, i.e. the size of the closed population in which the endemic takes place [20]. The transmission rate parameter *β* is a population specific constant that contains information on the contact rate and transmission probability between hosts [21].

In a genetically heterogeneous population, *β* varies between pairs of individuals, depending on the susceptibility genotype of the susceptible individual and the infectivity genotype of the infectious individual. We assumed that, between individuals, the susceptibility genotype and the infectivity genotype have independent effects, which is known as separable mixing in epidemiology [22], i.e., the susceptibility effect of individuals that are susceptible is independent of the infectivity effect of individuals that are infectious. Thus, the transmission rate parameter *β*_*ij*_ from an infectious individual with infectivity genotype *j* (*j* = *FF*, *Ff* or *ff*) to a recipient susceptible individual with susceptibility genotype *i* (*i* = *GG*, *Gg* or *gg*) was defined as:

$$\beta_{ij} = c\gamma_{i} \varphi_{j} ,$$

where *γ*_*i*_ is the susceptibility value for genotype *i* and $\varphi_{j}$ the infectivity value for genotype *j*. Without loss of generality, we chose *γ*_*g*_ = $\varphi_{f}$ = 1 as reference allele values. Therefore, *γ*_*gg*_ = $\varphi_{ff}$ = 1, so that *β*_*ggff*_ = *c*. Thus, *c* represents the transmission rate parameter from an infectious individual with infectivity genotype *ff* to a susceptible individual with susceptibility genotype *gg*. Since, *γ*_*g*_ = $\varphi_{f}$ = 1, *γ*_*G*_ represents the ratio of the value of allele *G* over the value of allele *g*, and $\varphi_{F}$ represents the ratio of the value of allele *F* over the value of allele *f*. For example, *γ*_*GG*_/*γ*_*Gg*_ = *γ*_*G*_, and *γ*_*Gg*_/*γ*_*gg*_ = *γ*_*G*_.

The *total* infectivity to which susceptible individuals are exposed at time *t,* depends on the total number of infectious individuals of each genotype at that time *I*_*j*_(*t*) and is measured by $\sum\nolimits_{j} {\left( {\varphi_{j} I_{j} \left( t \right)} \right)}$. Thus, the infection rate at time *t* for susceptible individuals with genotype *i* $\left( {Infection rate_{i} \left( t \right)} \right)$, depends on the susceptibility of genotype *i* and on the total infectivity of infectious group mates:

1 $$Infection\,rate_{i} \left( t \right) = c \gamma_{i} \frac{{S_{i} \left( t \right) }}{N}\mathop \sum \limits_{j} \left( {\varphi_{j} I_{j} \left( t \right)} \right),$$

where *S*_*i*_(*t*) is the number of susceptible individuals with genotype *i* at time *t*.

The probability per unit of time for an individual to recover and become susceptible again was given by the recovery rate parameter *α* and was assumed to be the same for all genotypes. Note that a single *α* does not imply the same infectious period for all individuals; because *α* is a stochastic rate, the length of the infectious period follows an exponential distribution and thus shows random phenotypic, albeit not genetic, variation among individuals.

### Generalized linear model

To estimate the effect of single SNPs on both host susceptibility and host infectivity, we developed a generalized linear model (GLM). The GLM was based on the infection rate given by Eq. (1). We assumed that the recording interval, the disease status of individuals at recording, and the genotypes of individuals were known.

For the sake of readability, the index *t* is dropped in the following and, hence, *S*, *S*_*i*_, *I*, and *I*_*j*_ refer to the number of individuals at the beginning of the interval. Then, the probability *P*_*i*_ for a single susceptible individual with genotype *i* to get infected when exposed to all infectious individuals during an interval $\Delta t$, follows from assuming a Poisson process within $\Delta t$. It is the probability of a non-zero outcome from a Poisson distribution, and follows from Eq. (1) with *S*_*i*_ = 1,

2 $$P_{i} = 1 - e^{{ - c\gamma_{i} \left( {\mathop \sum \limits_{j} \varphi_{j} I_{j} } \right)\Delta t/N}} .$$

The second term on the right-hand side is the zero-term of the Poisson distribution, which gives the probability of no infection. Thus, the number individuals with genotype *i* that become infected during $\Delta t$, i.e., cases *C*_*i*_, follows a binomial distribution with binomial total *S*_*i*_, i.e., depends on the number of susceptible individuals of genotype *i* at the start of the interval and the probability to become infected given by Eq. (2) [23]. Equation (2) assumes that infections are only caused by individuals that were infectious at the beginning of the interval (*I*_*j*_). In other words, the effect on the *P*_*i*_ of individuals that became infected or recovered during the interval is ignored in Eq. (2). This assumption is increasingly violated at longer recording intervals. Thus, we investigated the effect of the recording interval on the quality of the estimates and whether an optimum recording interval exists.

Because the probability to become infected follows from the complement of the zero-term of the Poisson distribution (Eq. 2), the complementary log–log is the appropriate link function to connect the explanatory variables to the expected value of the observed variable [17, 23]. Thus, a GLM with a complementary log–log link function was used to estimate effects of SNPs:

$$\begin{aligned} cloglog\left( {P_{i} } \right) & = log\left( { - log\left( {1 - P_{i} } \right)} \right) \\ & = log\left( c \right) + log\left( {\gamma_{i} } \right) + log\left( {\mathop \sum \limits_{j} \frac{{I_{j} }}{I}\varphi_{j} } \right) + log\left( {\frac{I}{N}\Delta t} \right), \\ \end{aligned}$$

where *I* is the total number of infected individuals at the beginning of the interval, such that $\frac{{I_{j} }}{I}$ represents the fraction of infectious individuals with infectivity genotype *j* at the beginning of the interval. As noted by Anche et al. [17], this model is linear in *log*(*γ*_*i*_) but not in *log*($\varphi_{j}$). To linearize the model, the arithmetic mean of $\varphi$, $\sum\nolimits_{j} {\frac{{I_{j} }}{I}} \varphi_{j}$, was approximated by the corresponding geometric mean, $\prod\nolimits_{j} {\varphi_{j}^{{\frac{{I_{j} }}{I}}} }$ [17], such that $log\left( {\sum\nolimits_{j} {\frac{{I_{j} }}{I}} \varphi_{j} } \right) \approx log\left( {\prod\nolimits_{j} {\varphi_{j}^{{\frac{{I_{j} }}{I}}} } } \right) = \sum\nolimits_{j} {\frac{{I_{j} }}{I}} log\left( {\varphi_{j} } \right)$. Now, the GLM is linear in both *log*(*γ*_*i*_) and *log*($\varphi_{j}$):

$$cloglog\left( {P_{i} } \right) \approx \log \left( c \right) + \log \left( {\gamma_{i} } \right) + \mathop \sum \limits_{j} \frac{{I_{j} }}{I}\log \left( {\varphi_{j} } \right) + \log \left( {\frac{I}{N}\Delta t} \right).$$

Details on the error caused by this approximation are in the appendix of [17], and are <5% for infectivity effects up to a factor of 3 (i.e., $\varphi_{F}$ between 0.33 and 3.0).

By assuming multiplicative allele effects on the original scale, allele effects were additive on the log-scale. For susceptibility, for example, *log*(*γ*_*gg*_) = 0, *log*(*γ*_*Gg*_) = *log*(*γ*_*G*_), and *log*(*γ*_*GG*_) = 2*log*(*γ*_*G*_). Thus, under this assumption, the model can be expressed in terms of allele counts [17]. Furthermore, we added a random group effect to account for possible additional (stochastic) differences in transmission between groups. When a random group effect is added to the model, the standard deviations of the estimated parameters are higher than those from a model without group included as a random effect. Although we did not simulate group effects in this study, they must be estimated in real data. Thus, we included a random group effect to better reflect the standard errors on the allele effect estimates that may be found in real data. A generalized linear mixed model (GLMM) allows for the inclusion of random effects resulting in the following final GLMM:

3 $$cloglog\left( {P_{i} } \right) = c_{0} + c_{1} CountG + c_{2} CountF + log\left( {\frac{I}{N}\Delta t} \right) .$$

where *c*_0_ = *log*(*c*) is the intercept. To achieve that *γ*_*g*_ = $\varphi_{f}$ = 1, such that ${ \log }(\gamma_{g} ) = { \log }\left( {\varphi_{f} } \right) = 0$, we counted alleles *G* and *F* within individuals, rather than alleles *g* and *f*, such that the regression coefficients represent the value of a single copy of allele *G* or *F*. For example, the ratio of *γ*_*G*_ versus *γ*_*g*_ is $\gamma_{G} = e^{{c_{1} }}$, which is estimated by $\hat{\gamma }_{G} = e^{{\widehat{{c_{1} }}}}$. Thus, *CountG* represents the number of *G*-alleles at the susceptibility locus of the susceptible individual, takes values 0, 1 or 2, and has coefficient $c_{1} = \log \left( {\gamma_{G} } \right).$ *CountF* represents the average number of *F*-alleles at the infectivity locus in the infected individuals, takes real values between 0 and 2, and has coefficient $c_{2} = { \log }\left( {\varphi_{F} } \right)$. *CountF* is calculated as $\frac{{2I_{FF} + I_{Ff} }}{I}$, where *I*_*FF*_ is the number of infected individuals with genotype *FF* at the beginning of the interval and *I*_*Ff*_ is the corresponding number of infected individuals with genotype *Ff*. The denominator of *CountF* is *I* rather than 2*I* because *CountF* is the average number of *F* alleles rather than its proportion. Table 1 summarizes the relationship between the regression coefficients of the GLMM and the transmission rate parameters for each genotype. The final model term, $log\left( {\frac{I}{N}\Delta t} \right)$, is a known offset, i.e., an “explanatory variable” with coefficient equal to 1. The time period *Δt* determines the interpretation of the transmission rate parameter. For example, rates are per day when the time period *Δt* is expressed in days.

**Table 1:** Relationship between the transmission rate parameters and the regression coefficients of the generalized linear mixed model for each genotype

Transmission rate parameter^a^	Expression in terms of regression coefficients
*β*_*ggff*_	$e^{{c_{0} }}$
*β*_*Ggff*_	$e^{{c_{0} + c_{1} }}$
*β*_*GGff*_	$e^{{c_{0} + 2c_{1} }}$
*β*_*ggFf*_	$e^{{c_{0} + c_{2} }}$
*β*_*GgFf*_	$e^{{c_{0} + c_{1} + c_{2} }}$
*β*_*GGFf*_	$e^{{c_{0} + 2c_{1} + c_{2} }}$
*β*_*ggFF*_	$e^{{c_{0} + 2c_{2} }}$
*β*_*GgFF*_	$e^{{c_{0} + c_{1} + 2c_{2} }}$
*β*_*GGFF*_	$e^{{c_{0} + 2c_{1} + 2c_{2} }}$

^a^The first two subscripts of *β* indicate the susceptible genotype of susceptible individuals, the second two subscripts indicate the infectivity genotype of infectious individuals. It follows that $\gamma_{G} = e^{{c_{1} }}$ and $\varphi_{F} = e^{{c_{2} }}$

### Simulations

To evaluate the quality of the estimates from the above model, we simulated an endemic disease and quantified bias and precision of SNP effects estimated based on Model 3. Bias was defined as the difference between the estimated and true effects of each SNP and relative bias was defined as the bias relative to the true size of the effect. Absolute bias and relative bias were calculated on the original scale. Precision was measured by the root mean squared error (RMSE) of the estimated SNP effects on the original scale. Simulations were conducted in R version 3.2.3. and data were analysed with the R-package lme4 [24, 25], using the glmer() function to solve the GLMM with Gauss-Hermite quadrature methods.

A group (defined as closed and random mixing) consisted of 100 individuals, which resembles for example, a dairy herd in the Netherlands. In dairy herds, a limited number of sires is used, so that cows in the same herd are (slightly) more related to each other than to cows in other herds. We simulated such genetic heterogeneity by sampling allele frequencies for susceptibility (*p*_*G*_ and *p*_*g*_ = 1 − *p*_*G*_), and infectivity (*p*_*F*_ and *p*_*f*_ = 1 − *p*_*F*_) for each group from a beta distribution with a mean of 0.5 and standard deviation of 0.05. We chose a beta distribution for *p* to ensure that allele frequencies are between 0 and 1. For the mean allele frequency, we used 0.5, which is simply the centre of the 0–1 interval. We assumed that the susceptibility effect of an individual and that same individual’s infectivity effect were not correlated. Within groups, genotypes were sampled assuming Hardy–Weinberg equilibrium. The loci for susceptibility and infectivity were simulated in linkage equilibrium.

Next, an initial disease status was modelled for each individual. Because interest was in obtaining data from the endemic phase of the disease, the endemic phase was started at the equilibrium in terms of number of susceptible and infectious individuals (details are in the “Appendix”). The next event, infection or recovery, was sampled using the direct method of the Gillespie’s algorithm [26], where the probability that a specific event occurred was proportional to the rate with which that event occurred (see [17] for an example). Thus, time between events was sampled from an exponential distribution with the sum of the rates of infection and recovery as parameter. If the endemic phase died out (no infectious individuals in the population), a random individual was infected immediately. This case was excluded from the analysed data, but included as explanatory variable in the model for subsequent cases.

One replicate consisted of 10 groups of 100 individuals each. In each replicate, individual disease status was recorded 11 times, and individual genotypes were known.

### Scenarios

Table 2 shows the input values for scenarios 1 and 2.

**Table 2:** Input values for the simulations

Variable	Scenario 1	Scenario 2
	SNP effect	Recording interval
Group size	100	100
Trans. rate par. ref. type (c)^a^	0.8–0.145	0.6
Recovery rate (*α*)	0.0476	0.0476
Average infectious period (1/*α*) [days]	21	21
Value susceptibility allele *g* (*γ*_*g*_)	1	1
Value susceptibility allele *G* (*γ*_*G*_)	0.3–1	0.4
Value infectivity allele *f* ($\varphi_{f}$)	1	1
Value infectivity allele *F* ($\varphi_{F}$)	0.3–1	0.4
Frequency allele *g* (*p*_*g*_)	Beta (0.5, 0.05)	Beta (0.5, 0.05)
Frequency allele *f* (*p*_*f*_)	Beta (0.5, 0.05)	Beta (0.5, 0.05)
Basic reproduction ratio (*R*_0_)	3.0	3.0
Endemic reproduction ratio (*R*)^b^	2.1–3.0	2.4
Recording interval (% of 1/*α*) [%]	66.6	4.8–133.3
Recording frequency	11 times (10 intervals)	11 times (10 intervals)

^a^Transmission rate parameter for the reference genotype *ggff*

^b^Details on the calculation of the endemic reproduction ratio are in the “Appendix”

In scenario 1, we varied *γ*_*G*_ and $\varphi_{F}$ simultaneously between 0.3 and 1, while keeping *γ*_*g*_ = $\varphi_{f}$ = 1, to investigate statistical power to identify SNP effects on susceptibility and infectivity. A value for *γ*_*g*_ = 0.3, for example, means that the *Gg* genotype is $1/0.3 = 3\frac{1}{3}$ times less susceptible than the *gg* genotype, while the *GG* genotype is 1/0.3^2^ = 11.1 times less susceptible than the *gg* genotype.

In scenario 2, we varied the recording interval while keeping the total number of recordings constant, in order to find the optimal recording interval. The recording interval ranged from 4.8 to 133.3% of the average infectious period (1/*α*). For all recording intervals in Scenario 2, *γ*_*G*_ = $\varphi_{F}$ = 0.4. To check whether the optimal recording interval depends on the effect size, we also investigated a scenario with *γ*_*G*_ = $\varphi_{F}$ = 0.6.

## Results

**Fig. 1:**
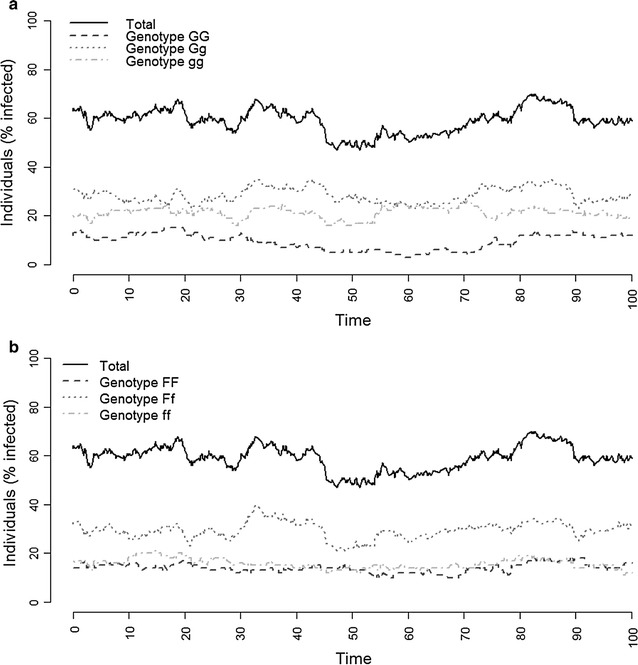
Percentage of infected individuals within a given susceptibility (**a**) and infectivity (**b**) genotype during 100 days of an endemic disease. Results are from one representative replicate with *p*_*g*_ = *p*_*f*_ = 0.5 and *γ*_*G*_ = $\varphi_{F}$ = 0.4

Estimates in this section are averages of 200 replicates, except for Fig. 1, which shows the result for one replicate. Infectivity estimates were not corrected for the geometric mean approximation because the error caused by this approximation was found to be small, as quantified (Tables 3, 5).

**Table 3:** Estimates of the effect of susceptibility, bias, precision, and power for different allele effect sizes

Input (*γ*_*g*_ − *γ*_*G*_)^a^	Estimate ($\gamma_{g} - \hat{\gamma }_{G}$)^a^	Bias		RMSE	Power (%)
		Absolute	Relative (%)		
0.0	−0.001	−0.001	−0.1	0.033	2
0.1	0.087	−0.013	−13.4	0.033	78
0.2	0.173	−0.027	−13.3	0.039	100
0.3	0.265	−0.035	−11.7	0.043	100
0.4	0.358	−0.042	−10.5	0.046	100
0.5	0.457	−0.043	−8.6	0.047	100
0.6	0.558	−0.042	−7.1	0.046	100
0.7	0.663	−0.037	−5.3	0.039	100

Precision was measured by RMSE and results are averages of 200 replicates

^a^ *γ*_*g*_ = 1

Figure 1 shows an example of the percentage of infected individuals for each susceptibility and infectivity genotype during 100 days of an endemic. The distribution of the susceptibility genotypes within the infected individuals differed from the genotype frequency for susceptibility in the whole population (Fig. 1a). The *gg* genotype was overrepresented in the infected individuals because this genotype had above-average susceptibility, while the *GG* genotype was underrepresented in the infected individuals. Most of infected individuals, however, had the *Gg* genotype, simply because there were more individuals with this genotype. An overview of genotype specific prevalences for different allele values is in Table 6 of the “Appendix”. The distribution of the infectivity genotypes within the infected individuals was similar to the genotype frequencies in the whole population, because the susceptibility and infectivity loci were unlinked and in linkage equilibrium (Fig. 1b).

In scenario 1, we varied *γ*_*G*_ from 1 to 0.3, so that the susceptibility effect, *γ*_*g*_ - *γ*_*G*_, varied from 0 to 0.7 (Tables 3, 4). Since *γ*_*g*_ = 1, the susceptibility value of the *G* allele ranged from 100 to 30% of that of the *g* allele. Tables 3 and 4 show the estimates of susceptibility and infectivity effects, bias, precision, and power for different allele effect sizes. All SNP effects were underestimated. As expected, absolute bias increased with absolute size of the effect, for both susceptibility and infectivity. However, relative bias decreased with absolute size of the effect. Precision was measured by the RMSE, with higher values indicating less precision. For infectivity, the RMSE was 4.3 to 6.6 times higher than for susceptibility. There was no clear relationship between RMSE and true size of the SNP effect. For susceptibility, power to detect a SNP effect, defined as the probability to find a significant effect given that it exists, i.e., the percentage of replicates with a significant SNP effect (*P* < 0.05), was 100% for all values of *γ*_*G*_, except for *γ*_*g*_ − *γ*_*G*_ = 0.1, for which power was 78%. For infectivity, power increased from 5% for $\varphi_{f} - \varphi_{F} = 0.1$ to 90.5% for $\varphi_{f}$ − $\varphi_{F}$ = 0.7.

**Table 4:** Estimates of the effect of infectivity, bias, precision, power, and error caused by the geometric mean approximation (GMA)

Input ($\varphi_{f}$ − $\varphi_{F}$)^a^	Estimate ($\varphi_{f} - \hat{\varphi }_{F}$)^a^	Bias		RMSE	Power (%)	GMA error^b^
		Absolute	Relative (%)			
0.0	−0.011	−0.011	−1.1	0.215	2.0	−0.0002
0.1	0.029	−0.071	−71.4	0.212	5.0	0.0001
0.2	0.125	−0.075	−37.5	0.191	10.5	0.0005
0.3	0.197	−0.103	−34.3	0.185	23.0	0.0008
0.4	0.279	−0.121	−30.2	0.203	44.0	0.0017
0.5	0.350	−0.150	−30.0	0.222	60.0	0.0029
0.6	0.449	−0.151	−25.2	0.200	80.0	0.0052
0.7	0.529	−0.171	−24.5	0.203	90.5	0.0082

Precision was measured by RMSE and results are averages of 200 replicates

^a^ $$\varphi_{f}$$ = 1

^b^ $$\hat{\varphi }_{F} - \hat{\varphi }_{{F_{corrected \, for \, GMA} }}$$

**Fig. 2:**
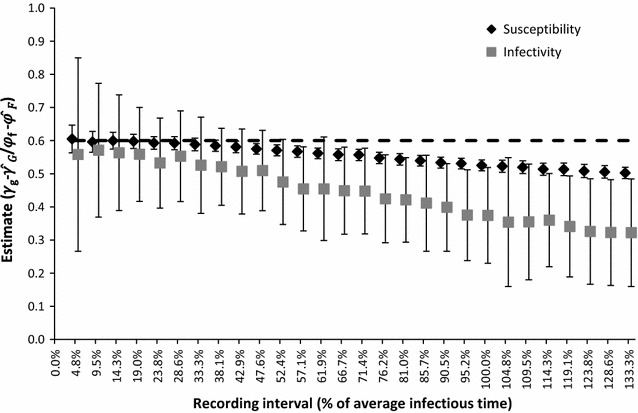
Susceptibility and infectivity estimates for different recording intervals. *Markers* show the estimates, which were averaged over 200 replicates. Input was *γ*_*g*_ − *γ*_*G*_ = $\varphi_{f}$ − $\varphi_{F}$ = 0.6 (*dashed line*). *Error bars* show the standard deviation among replicates on the original scale. Further inputs are in Table 2, Scenario 2

In Scenario 2, we varied the recording interval while keeping the total number of recordings constant. Figure 2 shows estimates of susceptibility and infectivity for different recording intervals. Table 5 shows the corresponding precision, power, and error caused by the geometric mean approximation (GMA). For all intervals, SNP effects were underestimated, except for the 4.8%-interval, for which the susceptibility effect was slightly overestimated, $\hat{\gamma }_{{G_{4.8\% } }} = 0.605$. Underestimation increased with length of the recording interval, which was more pronounced for infectivity. For susceptibility, bias was smallest (−0.04%) for the 14.3% interval, while for infectivity, bias was smallest (−4.8%) for the 9.5% interval. For susceptibility, power was 100% for all intervals, while precision was highest from the 25% interval to the 50% interval, and decreased for longer and shorter intervals. For infectivity, both power and precision were highest from the 25% interval to the 50% interval, and decreased for longer and shorter intervals. We found the same optimal recording interval for susceptibility and infectivity with *γ*_*G*_ = $\varphi_{F}$ = 0.6 (results not shown).

**Table 5:** Precision, power, and error caused by the geometric mean approximation (GMA) for different recording intervals

Recording interval % infectious time	RMSE		Power		GMA error^a^
	Susceptibility	Infectivity	Susceptibility (%)	Infectivity (%)	
4.8	0.042	0.294	100.0	47.0	0.0154
9.5	0.031	0.203	100.0	65.5	0.0133
14.3	0.025	0.178	100.0	75.5	0.0115
19.0	0.022	0.147	100.0	80.5	0.0105
23.8	0.020	0.152	100.0	80.5	0.0090
28.6	0.021	0.144	100.0	83.0	0.0088
33.3	0.022	0.163	100.0	84.5	0.0088
38.1	0.023	0.141	100.0	86.0	0.0080
42.9	0.026	0.158	100.0	84.0	0.0076
47.6	0.030	0.151	100.0	88.5	0.0074
52.4	0.034	0.179	100.0	85.5	0.0061
57.1	0.037	0.193	100.0	78.5	0.0053
61.9	0.042	0.213	100.0	81.5	0.0055
66.7	0.046	0.200	100.0	80.5	0.0052
71.4	0.046	0.200	100.0	82.5	0.0052
76.2	0.055	0.220	100.0	77.0	0.0045
81.0	0.059	0.219	100.0	79.5	0.0042
85.7	0.063	0.238	100.0	77.0	0.0041
90.5	0.069	0.241	100.0	75.5	0.0037
95.2	0.071	0.263	100.0	72.0	0.0032
100.0	0.076	0.268	100.0	65.0	0.0033
104.8	0.079	0.313	100.0	73.0	0.0028
109.5	0.083	0.301	100.0	68.5	0.0030
114.3	0.088	0.278	100.0	69.0	0.0028
119.1	0.089	0.301	100.0	66.0	0.0026
123.8	0.094	0.317	100.0	60.5	0.0024
128.6	0.096	0.320	100.0	66.0	0.0022
133.3	0.099	0.322	100.0	60.0	0.0023

Precision was measured by RMSE and results are averages of 200 replicates. Further inputs are in Table 2, Scenario 2

^a^ $$\hat{\varphi }_{F} - \hat{\varphi }_{{F_{corrected for GMA} }}$$

## Discussion

Given the potential importance of genetic effects on infectivity, we developed a model to estimate effects of host SNPs on both susceptibility and infectivity. The model accounts for variation among susceptible individuals in the exposure to infectious herd mates, and for the genotypes of those herd mates. To test our model, we simulated an endemic disease in 10 groups of 100 individuals and recorded time-series data on individual disease status. For different SNP effects and recording intervals, we quantified bias and precision of model estimates. SNP effects were on average underestimated, thus estimates were conservative. Underestimation of SNP effects on infectivity increased with length of the recording interval. In spite of the limited sample size simulated, power to detect SNP effects for susceptibility was high. Power to detect effects for infectivity was lower but became higher than 60% when the allele effect size was greater than a factor of 0.5. The optimal recording interval was similar for susceptibility and infectivity, around 25 to 50% of the length of the average infectious period.

In the development of our model, we followed Anche et al. [17], who considered epidemic diseases modelled by a SIR model. Given the importance of endemic diseases for livestock populations, we extended their approach to endemic diseases following a SIS model. Moreover, we considered time-series data on individual disease status, whereas Anche et al. [17] considered the final disease status of individuals after an epidemic had gone through the population. With time-series data, more information is available on who infected whom and on the variation among susceptible individuals in exposure to infectious herd mates. This increases the accuracy of SNP-estimates, particularly for infectivity [19]. We expect that our model can be easily extended to time-series data on epidemic diseases that follow a SIR model, because the underlying principle is the same. Each time-period can be treated as an incomplete epidemic, where the number of susceptible and infectious individuals at the beginning of the period and the number of cases during the period must be recorded.

Both susceptibility effects and infectivity effects were underestimated, which was more pronounced for longer recording intervals, likely because of unobserved infections and recoveries in-between the recording time points. Regarding underestimation of the susceptibility effect, a case is missed when a susceptible individual becomes infected and also recovers within the same time interval. Since recovery rate was the same for all genotypes, the probability to miss a case was higher for genotypes that are more susceptible. Hence, genotypes with higher susceptibility have a larger proportion of missed cases, which reduces the estimate of the susceptibility effect. Regarding underestimation of the infectivity effect, we used the number of infectious individuals of each genotype at the start of the time-interval, *I*_*j*_(*t*), as explanatory variable in our model. However, there is loss and gain of infectious individuals during the interval because on the one hand, some of the initially infectious individuals may recover during the interval and thus no longer contribute to transmission, while on the other hand, some of the initially susceptible individuals may become infected during the interval and contribute to transmission from that time onwards. This loss and gain of infectious individuals is not accounted for by the model, which is more pronounced for longer intervals. In a (dynamic) equilibrium, the number of infectious individuals will, on average, tend to move towards its median value. Hence, the number of infectious individuals of a certain genotype at the beginning of the interval is systematically more extreme than the actual number of infectious individuals of that genotype averaged over the interval. Thus, in the model, the variance of the *CountF*-term is systematically too large, especially for longer intervals. This explains the underestimation of the infectivity effect (i.e., *c*_2_) and the increase of this underestimation when the recording interval is longer. However, when the recording interval is short, there are no or only a few infections within an interval and, thus, the number of cases is too limited for precise estimations. Thus, given a fixed total number of recordings, short recording intervals lead to reduced precision of estimates, whereas long intervals lead to bias (Fig. 2). When the number of recordings is unlimited, the optimal recording interval will be short because the large number of records compensates for the limited precision of individual records but not for their bias.

An assumption of our model is that cases within an interval are caused by the infected individuals at the beginning of that interval. Thus, there is a gain and loss of infectious individuals that is not accounted for by the model. The impact of this error depends on the number of cases and the number of recoveries relative to the number of infected individuals at the beginning of the interval. In an endemic equilibrium, the number of cases within an interval equals, on average, the number of recoveries within an interval, *C* = *αI*. Hence, when expressed relative to the number of infected individuals at the beginning of the interval, the number of cases and the number of recoveries are both defined by the recovery rate *α*. Thus, the impact of the error caused by the assumption is determined by *α*, which suggests that the recovery rate (which equals the incidence in the endemic equilibrium) determines the optimum recording interval, rather than prevalence.

Estimates of genetic effects on infectivity were less accurate than those on susceptibility. This is partly because infectivity is expressed only by the infected individuals. Furthermore, there is a trade-off between the quality of the susceptibility and infectivity estimates in relation to group size [19]. In large groups, more information is available on the order in which individuals become infected, which leads to better susceptibility estimates, while in small groups it is easier to establish who infected whom, which leads to better infectivity estimates. Because large groups have multiple infected individuals at any given point in time, genetic differences in infectivity have to be estimated indirectly from the number of susceptible group mates that become infected and from the genotype fractions among the infected individuals at different points in time. Thus, especially in populations that consist of large groups, more records and groups are needed to estimate genetic effects on infectivity than on susceptibility.

We assumed that allele effects on susceptibility and infectivity were additive on the log-scale, such that the model could be formulated in terms of allele counts within individuals and the model could be tested without introducing estimation errors that might be present with additive allele effects on the original scale. Allele effects were, therefore, multiplicative on the original scale. With multiplicative allele effects, negative dominance is introduced on the original scale. The magnitude of the dominance relative to the additive effect, denoted as *d*/*a* following Falconer and Mackay [27] is:

$$\frac{d}{a} = \frac{{\gamma_{Gg} - 0.5\left( {\gamma_{gg} + \gamma_{GG} } \right)}}{{0.5\left( {\gamma_{gg} - \gamma_{GG} } \right)}},$$

with *γ*_*G*_ < *γ*_*g*_. So, for example, for a twofold effect with $\gamma_{G} = 0.5$ and *γ*_*g*_ = 1.0, the dominance deviation is one-third of the additive effect. For a tenfold effect, *d*/*a* = −0.81. Hence, in our model, alleles that cause a large increase in susceptibility or infectivity are almost completely recessive. Recessive alleles for susceptibility may be plausible because selection against recessive alleles with detrimental effects on fitness is inefficient, particularly when the frequency of the recessive allele is low. Hence, alleles that cause a large increase in susceptibility but are still segregating are probably recessive. Whether completely recessive alleles for infectivity are also plausible, is unknown at present.

An alternative perspective is that our model estimates the average effects of alleles on the log-scale, regardless of presence or absence of dominance on the log-scale. This is analogous to using ordinary additive models for estimating SNP effects, where the model captures the full average effect (*α*) of an allele, including the relevant dominance component (*α* = *a* + (*q* − *p*)*d*; [27]).

We determined $\hat{\alpha }$ for additive and multiplicative allele effects, to determine the impact on estimates when allele effects are additive on the original scale instead of multiplicative. Input values for the additive simulation were *γ*_*GG*_ = $\varphi_{FF}$ = 0.16, *γ*_*Gg*_ = $\varphi_{Ff}$ = 0.58, and *γ*_*gg*_ = $\varphi_{ff}$ = 1. So, with *p* = *q* = 0.5, the average effect *α* = 0.42 [27]. Estimates were $\hat{\gamma }_{G} = 0.45$ and $\hat{\gamma }_{F} = 0.63$, such that $\hat{\alpha } = \frac{{\hat{\gamma }_{gg} - \hat{\gamma }_{GG} }}{2} = \frac{{1 - 0.45^{2} }}{2} = 0.40$ for susceptibility and $\hat{\alpha } = 0.30$ for infectivity. For the multiplicative simulation, input values were *γ*_*GG*_ = $\varphi_{FF}$ = 0.16, *γ*_*Gg*_ = $\varphi_{Ff}$ = 0.4, and *γ*_*gg*_ = $\varphi_{ff}$ = 1, such that, with *p* = *q* = 0.5, the average effect *α* = 0.42. Estimates were $\hat{\gamma }_{G} = 0.44$ and $\hat{\gamma }_{F} = 0.55$, so $\hat{\alpha } = 0.40$ for susceptibility, and $\hat{\alpha } = 0.35$ for infectivity. This suggests that our model performs worse if allele effects are additive on the original scale instead of multiplicative.

We estimated the effect of two SNPs, one for infectivity and one for susceptibility, without fitting the effect of other genes that may affect these traits. This approach is similar to genome-wide association studies (GWAS) or candidate gene studies, where SNP effects are often fitted one at a time. Hence, the model presented here can be used as a starting point to explore and identify which loci affect the trait of interest. One approach could be to estimate both the susceptibility effect and the infectivity effect of the same SNP, one SNP at a time. This would imply full linkage disequilibrium (LD) between the susceptibility SNP and the infectivity SNP, because they are one and the same SNP. However, in contrast to GWAS for ordinary (“direct”) traits, this would not imply full confounding of the two effects, because they are expressed in phenotypes of distinct individuals. Nevertheless, both effects may be partially confounded because herd mates are usually related. Hence, for GWAS, further research is required to investigate the effect of LD between SNPs for susceptibility and infectivity. Note that, while we considered absence of LD between loci in the simulated data, the statistical model that we developed (Eq. 2) does not make this assumption, because SNP effects are simply fitted as fixed effects in our model. Thus, estimates of SNP effects represent *partial* regression coefficients and, therefore, account for LD. As in any single-SNP GWAS, there may be genes elsewhere in the genome that affect the same trait and show LD with the SNP of interest. Such genes would bias estimates of the SNP of interest. Hence, after an initial single-SNP GWAS, the significant SNPs should ideally be fitted simultaneously, in order to account for LD. Moreover, in GWAS studies, significance thresholds need to account for multiple testing to avoid many false positives, and GWAS studies need to take population stratification into account. For traits affected by direct effects only, stratification can be accounted for by including a random polygenic effect in the model, with a covariance-structure given by a genomic relationship matrix. For infectious disease data, that model would need to be extended with polygenic effects for infectivity of infected contact individuals [19]. The latter model may also be suitable for genomic prediction, where the purpose is to estimate breeding values of individuals, rather than single gene effects.

Anche et al. showed that relatedness within groups resulted in better estimates of susceptibility and infectivity [17]. When relatedness within groups is high, individuals with above/below average susceptibility will also have group mates with above/below average susceptibility, and individuals with above/below average infectivity will also have group mates with above/below average infectivity. Relatedness within groups, therefore, increases variation between groups, which improves the estimates [17]. However, results from the field of indirect genetic effects indicate that relatedness may lead to confounding of direct and indirect effects. For example, when groups consist of a single family, direct and indirect effects are fully confounded [28]. This result may extend to infectious disease data when loci for susceptibility and infectivity are in LD. Further research is needed to identify the optimal group structure with respect to relatedness for estimating genetic effects on susceptibility and infectivity.

Knowledge of the amount of genetic variation in infectivity is very limited at present. In general, natural selection has a tendency to exhaust heritable variation in traits related to individual fitness. Infectivity, however, is an indirect genetic effect, that affects disease status of other individuals rather than that of the individual itself. Natural selection targets such indirect genetic effects only in the presence of feed-back mechanisms, such as with kin and group selection [12]. Even in the presence of such feed-back mechanisms, selection on indirect genetic effects is weaker than on direct genetic effects [29]. Thus, infectivity may have been less exposed to natural selection and may exhibit more genetic variation. Presence of genetic variation is also suggested by the existence of super spreaders [30]. The model presented here can be used as a starting point to determine the amount of genetic variation that is present for infectivity in populations. This may also help to better estimate effects on susceptibility because the model accounts for variation among susceptible individuals in their exposure to infectious herd mates and for the genotypes of those herd mates.

When our model is extended with the relevant polygenic effects (as discussed previously), it can be used to estimate SNP effects on susceptibility and infectivity, in particular when more data on disease status and genotype become available. Opportunities to measure disease status on a regular basis lie in the increasing number of sensor systems that are used and will be used in the future [31]. Current sensor systems are able to record animal activity, temperature, cells in milk, etc. In the future, these types of sensor data may provide regular information about the disease status of an animal. In addition, the number of animals that are genotyped increases rapidly. Combining the model developed here with genotype and sensor data may considerably enhance breeding against infectious diseases in livestock.

## Conclusions

We developed a generalized linear mixed model to estimate SNP effects on both host susceptibility and host infectivity from time-series data on individual disease status for an endemic disease. In contrast to common models used in animal breeding, our model accounts for variation among susceptible individuals in their exposure to infectious herd mates and for the genotypes of those herd mates. With the use of simulated data, we quantified bias and precision of SNP effects estimated by the model and showed that the optimal recording interval is between 25 and 50% of the average infectious period when disease status is observed 11 times. When the recording interval was close to optimal, SNP effects were on average slightly underestimated. Infectivity estimates were less precise than susceptibility estimates. In future genome-wide association studies, the model presented here may be useful to estimate SNP effects that affect disease transmission and disease prevalence.

## Appendix: Distribution of susceptible genotypes in the endemic equilibrium

In the absence of heterogeneity, the expected equilibrium prevalence follows from the basic reproduction ratio *R*_0_, and can be calculated as $1 - \frac{1}{{R_{0} }}$ [32, 33]. *R*_0_ can be expressed as a function of average susceptibility $\bar{\gamma }$, average infectivity $\bar{\varphi }$, the transmission rate parameter of the reference genotypes *c*, and the recovery rate parameter *α*, $R_{0} = \bar{\gamma }\bar{\varphi }\frac{c}{\alpha }$ [10]. Average susceptibility was calculated as $\bar{\gamma } = \sum\nolimits_{i} {p_{i} } \gamma_{i}$, and average infectivity as $\bar{\varphi } = \sum\nolimits_{j} {p_{j} } \varphi_{j}$, where *i* indexes susceptibility genotypes, *j* indexes infectivity genotypes, and *p*_*i*_ is the frequency of genotype *i* in the population.

However, with heterogeneity the equilibrium prevalence differs from the above result. At the start of an endemic phase, only few individuals are infected. Therefore, the *susceptible* fraction with susceptibility genotype *i* $\left( {fracS_{i} = \frac{{S_{i} }}{S}} \right)$ is similar to the genotype frequency of susceptibility genotype *i* (*p*_*i*_) in the population, *fracS*_*i*_ ≈ *p*_*i*_. In the endemic equilibrium, however, highly susceptible individuals have more chance to get infected, so the *susceptible* fraction with susceptibility genotype *i* differs from the genotype frequency of susceptibility genotype *i* in the population, *fracS*_*i*_ ≠ *p*_*i*_. Thus, in the endemic equilibrium, the average susceptibility of the susceptible individuals is lower than the average susceptibility in a totally susceptible population, therefore, the average susceptibility equals:

4 $$\bar{\gamma }\left( t \right) = \mathop \sum \limits_{i} fracS_{i} \left( t \right)*\gamma_{i} .$$

A lower average susceptibility in the equilibrium leads to a lower reproduction ratio and, therefore, a lower equilibrium prevalence as expected from the initial reproduction ratio. The reproduction ratio at time *t*, *R*(*t*), in a population that is no longer fully susceptible is given by:

5 $$R\left( t \right) = \bar{\gamma }\left( t \right)*\bar{\varphi }* \frac{c}{\alpha } .$$

Because the susceptibility locus and the infectivity locus were in linkage equilibrium, the *infected* fraction with infectivity genotype *j* $\left( {fracI_{j} = \frac{{I_{j} }}{I}} \right)$ will be similar to the total fraction with infectivity genotype *j* in the population, *fracI*_*j*_ ≈ *p*_*j*_.

As we approach the equilibrium, the *susceptible* fraction with susceptibility genotype *i* at time *t* + 1 can be calculated from the *susceptible* fraction with susceptibility genotype *i* at time *t*, and the corresponding *R*(*t*), by:

6 $$fracS_{i} \left( {t + 1} \right) = \frac{{p_{i} }}{{\frac{1}{R\left( t \right)} + \frac{{\gamma_{i} }}{{\bar{\gamma }}}\left( {1 - \frac{1}{R\left( t \right)}} \right)}} .$$

By using Eqs. (4–6) in an iterative process, the *susceptible* fraction with susceptibility genotype *i* in the endemic equilibrium was found. In the endemic equilibrium three conditions were met:

(i) $$\frac{{S_{i} }}{N} = \frac{1}{R}fracS_{i}$$ for *R* > 1
(ii) $$\frac{{I_{i} }}{N} = \left( {1 - \frac{1}{R}} \right)fracS_{i} \frac{{\gamma_{i} }}{{\bar{\gamma }}}$$ for *R* > 1
(iii) $$\frac{{S_{i} + I_{i} }}{N} = p_{i}$$.

Therefore, the genotype specific prevalences were known [conditions (i) and (ii)]. The basic reproduction ratio, the reproduction ratio in the equilibrium and the genotype-specific prevalences for different effects are in Table 6.

**Table 6:** Basic reproduction ratio and prevalence for different susceptibility effects

Value susceptibility allele G (*γ*_*G*_)	Transmission rate parameters for reference type (*c*)^a^	Basic reproduction ratio^b^		Prevalence			
				Total	Per susceptibility genotype		
		Classic ($R_{0}$)	Equilibrium ($R$)		$GG$	$Gg$	$gg$
0.3	0.8	3.00	2.10	0.52	0.25	0.53	0.79
0.4	0.6	3.03	2.39	0.58	0.36	0.59	0.78
0.5	0.45	3.00	2.59	0.61	0.45	0.62	0.77
0.6	0.35	3.01	2.77	0.64	0.52	0.64	0.75
0.7	0.28	3.07	2.94	0.66	0.58	0.66	0.74
0.8	0.22	3.03	2.98	0.66	0.61	0.67	0.71
0.9	0.18	3.08	3.07	0.67	0.65	0.67	0.70
1.0	0.145	3.045	3.045	0.67	0.67	0.67	0.67

^a^Reference genotype is ggff

^b^ *p*_*g*_ = *p*_*f*_ = 0.5, *α* = 0.0476, $$\gamma_{g} = \varphi_{f} = 1$$ and $$\varphi_{F} = \gamma_{G}$$

In this study, we started the endemic in the equilibrium. The distribution of the *infected* fraction of susceptibility genotypes in endemic equilibrium was obtained by a grid search for the point where conditions (i), (ii) and (iii) were met (note that the fastest way to reach the equilibrium goes through fractions that are in reality not possible, i.e., the path to the equilibrium is not real).
